# Preparation of P3HB4HB/(Gelatin + PVA) Composite Scaffolds by Coaxial Electrospinning and Its Biocompatibility Evaluation

**DOI:** 10.1155/2017/9251806

**Published:** 2017-11-19

**Authors:** Min-Xian Ma, Qin Liu, Chuan Ye, Brian Grottkau, Bing Guo, Yu-Feng Song

**Affiliations:** ^1^Guizhou Medical University, Guiyang 550004, China; ^2^Department of Prosthodontics, The Affiliated Hospital of Guizhou Medical University, Guiyang 550004, China; ^3^Department of Orthopaedics, The Affiliated Hospital of Guizhou Medical University, Guiyang 550004, China; ^4^Center for Tissue Engineering and Stem Cells, Guizhou Medical University, Guiyang 550004, China; ^5^Department of Orthopaedics, Massachusetts General Hospital, Boston, MA 02115, USA

## Abstract

This study was conducted to prepare coaxial electrospun scaffolds of P3HB4HB/(gelatin + PVA) with various concentration ratios with P3HB4HB as the core solution and gelatin + PVA mixture as the shell solution; the mass ratios of gelatin and PVA in each 10 mL shell mixture were 0.6 g : 0.2 g (Group A), 0.4 g : 0.4 g (Group B), and 0.2 g : 0.6 g (Group C). The results showed that the pore size, porosity, and cell proliferation rate of Group C were better than those of Groups A and B. The ascending order of the tensile strength and modulus of elasticity was Group A < Group B < Group C. The surface roughness was Group C > Group B > Group A. The osteogenic and chondrogenic-specific staining showed that Group C was stronger than Groups A and B. This study demonstrates that when the mass ratio of gelatin : PVA was 0.2 g : 0.6 g, a P3HB4HB/(gelatin + PVA) composite scaffold with a core-shell structure can be prepared, and the scaffold has good biocompatibility that it may be an ideal scaffold for tissue engineering.

## 1. Introduction

Electrospinning is an emerging technology for the preparation of tissue engineering scaffolds. Because of its unique structure, electrospun nanofibers exhibit excellent properties, including a high specific surface area and high porosity. Coaxial electrospinning is an effective method for the preparation of hollow nanofibers and tissue engineering scaffolds, and the core and shell materials can be selected judiciously to meet the requirements for various applications [1]. Without question, the construction of tissue engineering scaffolds and the selection of the scaffold material are inseparable, with the scaffold material being fundamental to tissue engineering. Natural polymer materials are derived from organisms and can eventually degrade into polysaccharides or amino acids for absorption by the body, assuming cell and tissue compatibility. However, their mechanical properties are poor, and their degradation is not easy to control. Synthetic polymer materials have good controllable physical and mechanical properties, degradation, and strength, yet their biocompatibility is poor [2]. Clearly, these two types of materials have unique advantages and disadvantages in terms of mechanical properties, biocompatibility, hydrophilicity, and electrospinning performance. To prepare an ideal scaffold material, it is clearly desirable to develop a composite scaffold material with select characteristics of distinct materials to benefit from their inherent advantages [3–6].

P3HB4HB is a fourth-generation biodegradable polymer in the PHA family. In addition to its excellent biocompatibility, biodegradability, and electrospinning performance, P3HB4HB also has good mechanical properties; thus, it possesses a wide range of potential applications in tissue engineering. However, P3HB4HB has poor hydrophilicity, which limits its application in regenerative medicine [7, 8]. Gelatin is a protein derived from partially denatured collagen and is a hydrophilic natural polymer material with good biocompatibility [9–11]. The composite construction of PHA and gelatin can improve the hydrophilicity of PHA and promote cell adhesion. Because gelatin requires heat to dissolve, it will coagulate into a colloid, and the electrospinning performance will decrease when the temperature decreases [12]. Therefore, in this study, polyvinyl alcohol (PVA), which possesses good spinnability, was added to the gelatin aqueous solution to improve its electrospinning performance. PVA and gelatin are both water-soluble polymers [13–15]. Overall, the good electrospinning performance of PVA and the good hydrophilicity of gelatin makes their mixture as the shell solution in coaxial electrospinning potentially useful to improve the poor hydrophilicity of P3HB4HB.

Three types of materials with different properties were used for coaxial electrospinning to prepare tissue engineering composite materials with a core-shell nanostructure and with the advantages of both natural and synthetic materials. The physical and chemical properties and cell compatibility of the scaffolds were evaluated to determine the optimal concentration ratio to meet the tissue engineering requirements.

## 2. Materials and Methods

### 2.1. Materials

The main materials and equipment used in this study include P3HB4HB (91% of 3HB and 9% of 4HB, MW = 280000, Tsinghua University Institute of Polymer); polyvinyl alcohol (PVA, MW = 186000, Tsinghua University Institute of Polymer); gelatin (GEL, Sigma, Type A); an electrospraying unit (SS-2534H, Ucalery, Beijing, China); an electron microscope (Hitachi, Japan); a universal test machine (MTS System Corporation, China); an incubator (Thermo, China); and a static contact angle measurement system (JC2000C type, Shanghai Zhongchen Digital Technic Apparatus Co., China).

### 2.2. Preparation of the P3HB4HB/(GE + PVA) Nanofiber Scaffold

P3HB4HB dissolved in dichloromethane, gelatin and PVA dissolved in deionized water, a 6% w/v P3HB4HB solution was prepared as the core solution of coaxial electrospinning, and an 8% w/v gelatin + PVA mixed solution was prepared as the shell solution, in which the mass ratios (in g) of gelatin to PVA in every 10 mL mixed solution were 0.6 : 0.2 (Group A), 0.4 : 0.4 (Group B), and 0.2 : 0.6 (Group C). The P3HB4HB and gelatin + PVA solutions were extracted using a 5 mL syringe with P3HB4HB in the inner tube and gelatin + PVA in the outer tube. A coaxial nozzle was also connected; the needle tip is connected to the high voltage DC (direct current) power supply. The coaxial electrospinning scaffold was prepared with a positive voltage of 17 kV, a negative voltage of 2.5 kV, a receiving distance of 30 cm (from coaxial nozzle to the receiver), and a solution injection rate of 1 mm/min.

### 2.3. Pore Size and Porosity of the Nanofiber Scaffold

According to the principle of mercury intrusion [16, 17], mercury has no wettability to most solid materials, and it needs additional pressure to enter the solid hole. For the pore model, the size of the hole that mercury can enter and pressure conforms to the Washburn equation. The smaller the aperture, the greater the required pressure, and the external pressure is inversely proportional to the net value of the amount of mercury injected. The relationship between pressure and aperture *d* = (10 × 4*γ*cos⁡*θ*)/*p*. In this formula, *d* is the diameter of the hole; *P* is the pressure of entering mercury; *γ* is the surface tension of liquid mercury; *θ* is the contact angle of liquid mercury and material. The scaffold porosity was measured by a modified liquid displacement method [18]. The specific procedures include the following: the anhydrous ethanol in volume *V*1 was added to a graduated test tube; the dry scaffold sample was cut into the appropriate size and immersed in ethanol for 5 min; the solution was degassed by vacuum until no bubbles were released from the scaffold, and the volume of ethanol in which the scaffold was immersed was recorded as *V*2; the scaffold sample was gently removed, and the volume of the remaining ethanol was measured as *V*3. The porosity of the scaffold was calculated according to the following equation: [(*V*1 − *V*3)/(*V*2 − *V*3)] × 100%. Three samples in each group were measured, and their average was used as the reported value.

### 2.4. Surface Roughness Experiments

Three groups of samples were cut into 1 cm × 1 cm size. The test pieces of Mitutoyo (SJ-310) were slid on the samples at a speed of 0.5 mm/s. The detection length was 0.5 cm. Each group of fiber membranes was tested for 5 samples, taking the mean.

### 2.5. Measurement of the Contact Angle

The samples in the three groups were cut into 1 cm × 1 cm pieces, spread flat on slides, and then fixed. At room temperature, 5 *μ*L of deionized water was taken with a microsyringe and dropped onto the sample surface. After standing for 5 s, the angle between the tangent of the deionized water droplet and the plate was measured, and the value of the contact angle was recorded. Each sample was measured at 5 different positions.

### 2.6. Mechanical Test

The materials in the three sample groups were cut into 60 mm × 15 mm pieces, with an effective stretch length of 40 mm. The universal electronic testing machine (Meters Industrial Systems, Inc., China) was used to measure the tensile test (with a 100 N sensor and a tensile speed of 5 mm/min). The tensile strength, Young's modulus, and nominal strain fracture of the scaffolds were obtained by averaging the values from five samples.

### 2.7. *In Vitro* Degradation Experiments

First, membranes of the three groups were cut into 1 cm × 1 cm, placed in the 60°C drying oven for 2 h, and weighed and recorded as *M*0, and then the membranes were put into small beaker which contains 10 mL saline in it, and this small beaker was put into 37°C constant temperature water tank and taken out in 1 d, 3 d, 5 d, and 7 d; the water on the surface of the membranes is absorbed with filter paper, and then they were placed in the 60°C drying oven for 2 h, weighed, and recorded as *M*1. So the degradation of the composite membrane = [(*m*0 − *m*1)/*m*0] × 100%; repeat the above steps twice; the average of the results of the 3 times is the dissolved rate of composite membrane.

### 2.8. Isolation and Culture of Human Bone Marrow Mesenchymal Stem Cells (hBMSCs)

hBMSCs were isolated from human bone marrow obtained from aspirates collected under informed consent from patients (all donors have been informed about the trial and given their consent). The aspirates were centrifuged at 1400 r/s for 5 min to obtain a high-density cell pellet, and the supernatant was removed. The cells were resuspended according to a 1 : 1 proportion with the culture medium (i.e., Dulbecco's modified eagle medium (DMEM) supplemented with 10% fetal bovine serum (FBS), 100 units/mL penicillin, and 100 mg/mL streptomycin) and then placed in cell culture flasks at a density of 3 × 10^6^ cells/mL in an incubator at 37°C and 5% CO_2_. The cells were passaged by treatment with a 0.2% trypsin solution when the cell confluence reached 80%. The fifth-passage cells were used for the subsequent experiments.

### 2.9. Cell Seeding and* In Vitro* Multilineage Differentiation

The three scaffold types were prewetted with DMEM. The fifth-passage hBMSCs were pipetted onto the three scaffolds at a cell density of 1 × 10^7^/scaffold and then cultured in an incubator at 37°C and 5% CO_2_ for 2 days. The culture medium was then replaced with a chondrogenic medium containing DMEM, 1% FBS, 10 ng rh-TGFb1/mL, 50 mg ascorbic acid/L, 6.25 mg insulin/mL, and 10^−7^ M dexamethasone and an osteogenic medium containing 10 mM b-glycerophosphate, 0.1 M dexamethasone, 50 g L-ascorbic acid 2-phosphate/mL, and 10 g insulin/mL. The medium was replaced every 3 days. Other cell/scaffold constructs were also prepared and incubated with a control medium.

The cell/scaffold constructs were fixed and assessed at different time points after multilineage differentiation. Following the manufacturer's instructions, the staining of alizarin red S, alkaline phosphatase (ALP), safranin O, and alcian blue (BCIP/NBT staining kit, Bi Yuntian, China) were then performed to ascertain osteogenic and chondrogenic differentiation.

### 2.10. Cell Adhesion Test

The three groups of scaffolds were cut into the size of 24-well plates, and then the resuspended fourth-generation hBMSCs were inoculated on the scaffold (the number of inoculated cells recorded as *D*0). hBMSCs-scaffolds complex was cultured at 1, 3, and 6 h, and the culture medium from 5 holes was taken out each time; the number of cells in the medium (*D*1) and attached cells was digested by trypsin and calculated as *D*2 (cell adhesion rate = [(*D*0 − *D*1 − *D*2)/*D*0] × 100%).

### 2.11. Cell Proliferation Assay

The three groups of scaffolds were cut into 5 mm × 5 mm pieces and placed into 24-well plates. Fourth-generation hBMSCs were seeded onto the scaffolds as the experimental group at a cell density of 3 × 10^5^ cells/well. For the control group, the cells were directly seeded into a 24-well plate. At days 1, 3, 5, and 7, four samples were used, and 10 *μ*L of the CCK8 reagent was added in each well to culture in an incubator for 4 hours. Subsequently, 100 *μ*L of the medium from each well was added to a 96-well plate, and the absorbance value at 450 nm was measured.

### 2.12. Scanning and Transmission Electron Microscopy

At day 7 after seeding, the cell/scaffold constructs were washed with phosphate-buffered saline (PBS) and fixed in 2.5% glutaraldehyde in PBS overnight at 4°C. The constructs were then stained with 1% osmium tetroxide, dehydrated in a gradient series of alcohol, freeze-dried for 8 h, and coated with gold.

The morphological structures of the different scaffolds were observed with a scanning electron microscope (SEM) at an accelerating voltage of 18 kV. Place the scaffolds in deionized water at room temperature for 24 h and 48 h; the change of fiber structure was observed by SEM. Transmission electron microscope (TEM) was used to visualize the core-shell structure of the composite fibers. 100 fibers were randomly selected from each group for fiber diameter analysis.

### 2.13. DAPI Staining

The cells were added dropwise to the nanofiber membrane, and the cells were cocultured with the nanofibers for 7 days. After the medium was removed, the cells were immobilized with ethanol for 5 min. DAPI (4′,6-diamidine-2′-phenylindole dihydrochloride) dye was added for staining at room temperature in the dark for 5–10 min, followed by a quick PBS wash (for a few seconds each time) in triplicate. The results were observed using fluorescence microscopy (with an excitation wavelength of 360–400 nm).

### 2.14. Statistical Analysis

All of the results are presented as the mean ± SD. The statistical comparisons were performed using the Student-Newman-Keuls (SNK) multiple-range test or the Kruskal-Wallis test followed by the Mann–Whitney *U* test (SPSS 17.0). Statistical significance was defined using a *P* value of <0.05; obviously statistical significance was defined using a *P* value of <0.01.

## 3. Results

### 3.1. General Appearance of the Nanofiber Scaffolds

**Figure 1:**
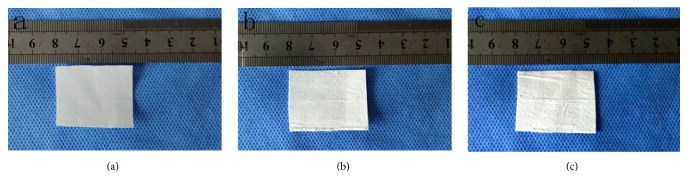
General appearance of the P3HB4HB/(GEL + PVA) scaffolds. (a) GEL : PVA = 0.6 g : 0.2 g; (b) GEL : PVA = 0.4 g : 0.4 g; (c) GEL : PVA = 0.2 g : 0.6 g.

The P3HB4HB/(GEL + PVA) nanofiber membrane was white, opaque, and uniform in thickness, with a certain strength and toughness. There was no significant difference in the appearance among the three groups (Figure 1).

### 3.2. Pore Size and Porosity of the Scaffolds

The pore sizes of the Group A, Group B, and Group C scaffolds were 60 ± 16 *μ*m, 68 ± 15 *μ*m, and 43 ± 5 *μ*m, respectively. The porosity of the Group A, Group B, and Group C scaffolds was 50.6%  ±  2.0%, 73.6%  ±  1.1%, and 81.6%  ±  1.6%, respectively. The pore size and porosity of Group C were significantly different compared with Groups A and B.

### 3.3. Surface Roughness Experiments

**Figure 2:**
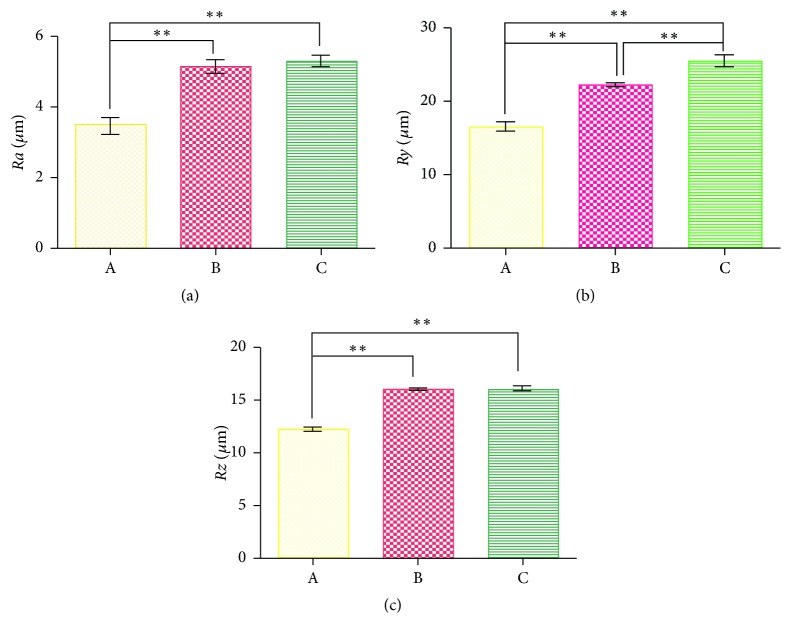
Surface roughness of the P3HB4HB/(GEL + PVA) scaffolds. (a) Roughness profile (*Ra*); (b) roughness height (*Rz*); (c) distance between contour peak line and contour bottom line (*Ry*). A: GEL : PVA = 0.6 g : 0.2 g; B: GEL : PVA = 0.4 g : 0.4 g; C: GEL : PVA = 0.2 g : 0.6 g. ^*∗∗*^*P* < 0.01.

Three groups of fiber membrane surface roughness of the main indicators: roughness profile (*Ra*), the roughness height (*Rz*), and the distance between the contour peak line and the contour bottom line (*Ry*). The results showed that, relative to A, the fiber in Groups B and C is more rough (*P* < 0.01, Figure 2).

### 3.4. Measurement of the Contact Angle

**Figure 3:**
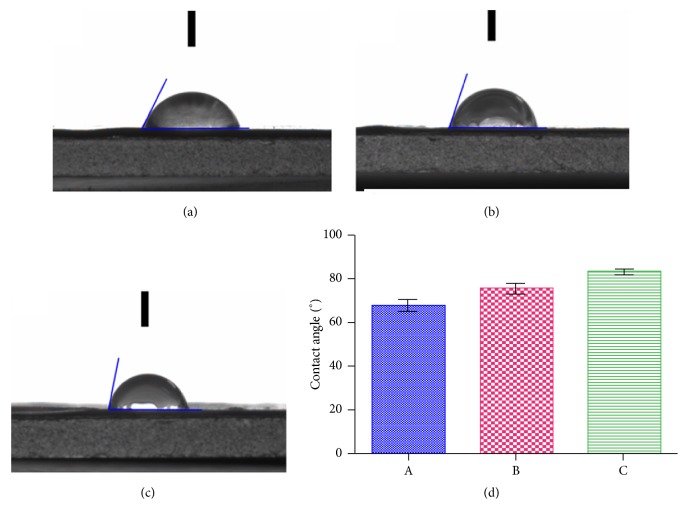
Contact angles of the P3HB4HB/(GEL + PVA) scaffolds. (a) GEL : PVA = 0.6 g : 0.2 g; (b) GEL : PVA = 0.4 g : 0.4 g; (c) GEL : PVA = 0.2 g : 0.6 g. A: GEL : PVA = 0.6 g : 0.2 g; B: GEL : PVA = 0.4 g : 0.4 g; C: GEL : PVA = 0.2 g : 0.6 g.

The contact angles at five different sites of the fiber membrane gave values of 68.125° ± 2.839°, 75.750° ± 2.630°, and 83.625° ± 1.315° for the Group A, Group B, and Group C scaffolds, respectively. The contact angle of Group A was smaller than those of Groups B and C, whereas the contact angle of Group B was smaller than that of Group C. With the decrease of the gelatine content in the electrospinning solution, the contact angle of the sample increased gradually, but they were all less than 90°, indicating hydrophilicity (Figure 3).

### 3.5. Mechanical Test

**Figure 4:**
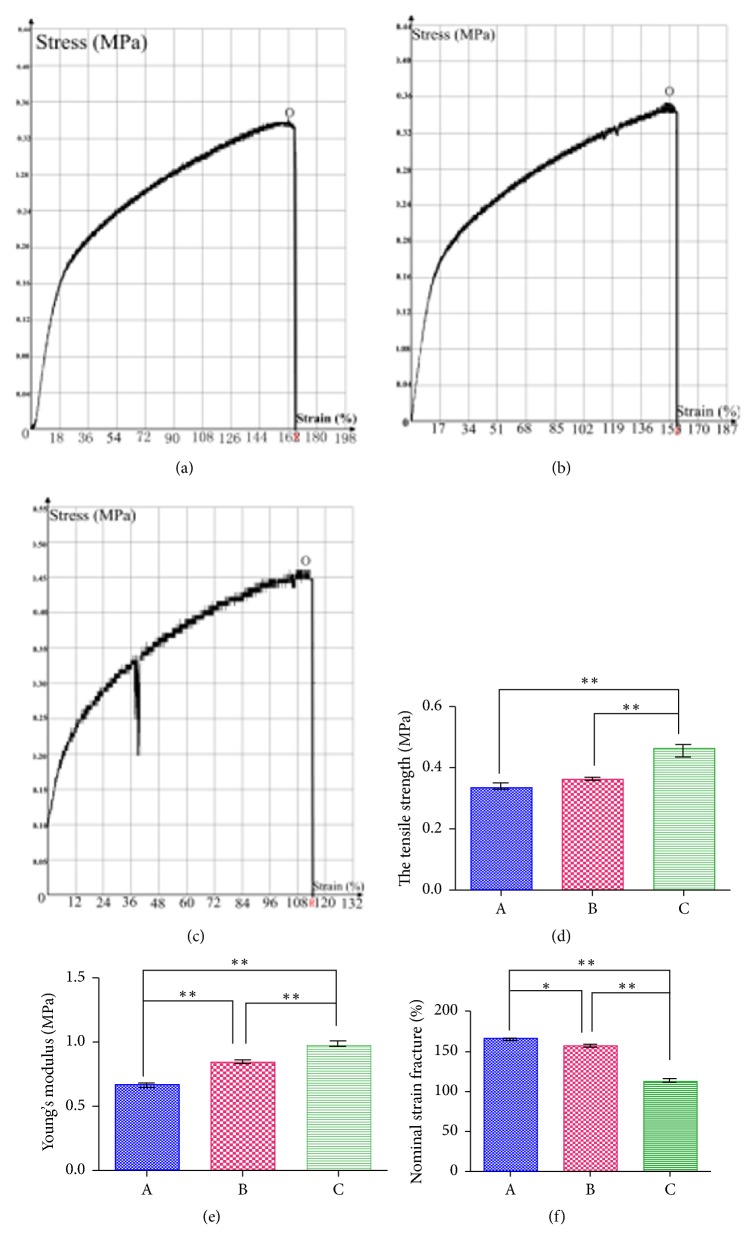
Stress-strain test and biomechanical properties of the P3HB4HB/(GEL + PVA) scaffolds. (a) Stress-strain test of GEL : PVA = 0.6 g : 0.2 g. (b) Stress-strain test of GEL : PVA = 0.4 g : 0.4 g. (c) Stress-strain test of GEL : PVA = 0.2 g : 0.6 g. (d) Tensile strength. (e) Young's modulus. (f) Nominal strain fracture. A: GEL : PVA = 0.6 g : 0.2 g; B: GEL : PVA = 0.4 g : 0.4 g; C: GEL : PVA = 0.2 g : 0.6 g. ^*∗*^*P* < 0.05; ^*∗∗*^*P* < 0.01.

Regarding the mechanical characterization of the electrospun composite nanofiber membranes (Figure 4), the stress at the highest point O is the maximum stress on the unit area of the material which is the tensile strength. The strain corresponding to point R is the nominal strain of the scaffold. The tensile strength and the modulus of elasticity increased as the gelatin content decreased, whereas the nominal strain fracture of Group A is the smallest. The tensile strength and the modulus of elasticity increased as the gelatin content decreased, whereas the nominal strain fracture decreased.

### 3.6. *In Vitro* Degradation Experiments

**Figure 5:**
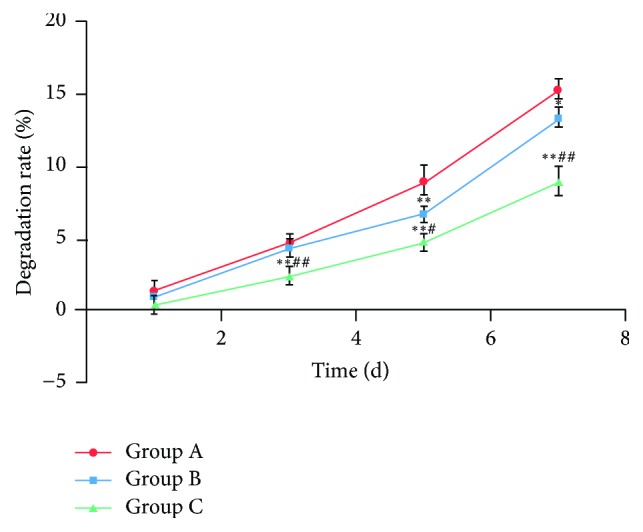
*In vitro* degradation of the P3HB4HB/(GEL + PVA) scaffolds on days 1, 3, 5, and 7 d. Group A: GEL : PVA = 0.6 g : 0.2 g; Group B: GEL : PVA = 0.4 g : 0.4 g; Group C: GEL : PVA = 0.2 g : 0.6 g. Compare to Group A; ^*∗*^*P* < 0.05; ^*∗∗*^*P* < 0.01; compare to Group B; ^#^*P* < 0.05; ^##^*P* < 0.01.

The degradation of fiber scaffolds increased with the prolongation of time, the degradation of group A was the fastest, and the degradation of group C was the slowest. With the prolongation of soaking time, the outer layer of nanofibers with gelatin gradually degrades; the weight of the scaffolds gradually decreases. Therefore, the degradation of group A with more gelatin was the highest, and the degradation of group C was the lowest (Figure 5).

### 3.7. Scanning Electron Microscopy

**Figure 6:**
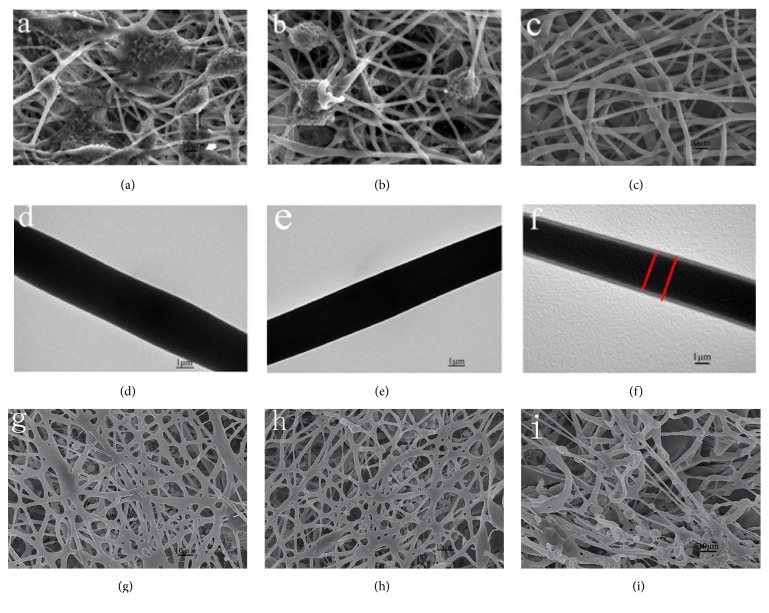
Scanning electron microscopy and transmission electron microscopy of the P3HB4HB/(GEL + PVA) scaffolds. (a, d) GEL : PVA = 0.6 g : 0.2 g; (b, e) GEL : PVA = 0.4 g : 0.4 g; (c, f) GEL : PVA = 0.2 g : 0.6 g. (g, h, i) GEL : PVA = 0.2 g : 0.6 g for the scaffolds not soaked in water, soaked for 24 h, and soaked for 48 h.

The scaffold materials were observed by SEM. The results indicate that the electrospinning of scaffold Groups A and B was poor, with significant beaded and honeycomb covering. Further, the fibers were randomly and disorderly arranged and intertwined, and the diameters of the fibers varied. In contrast, the Group C scaffold demonstrated an interconnected three-dimensional network structure. Moreover, the fiber surface was smooth and free of voids, and the diameters were relatively uniform (Figures 6(a), 6(b), and 6(c)). In Groups A and B, no obvious core-shell structure was observed, whereas a core-shell structure was observed in Group C using the projection electron microscope, and its core and shell were tightly connected (Figures 6(d), 6(e), and 6(f)). Place the scaffolds in deionized water for 24 h; the scaffolds structure and fibers have no significant changes (Figure 6(h)). After soaking for 48 h, as the gelatin partially dissolves, the fibers diameter becomes smaller and the pore size of scaffolds increased, but the scaffolds can keep their fiber structure (Figure 6(i)). The average diameter of three groups of fibers was 2.7 ± 0.34 *μ*m, 2.6 ± 0.21 *μ*m, and 2.6 ± 0.17 *μ*m, respectively.

### 3.8. Cell Adhesion Test

**Figure 7:**
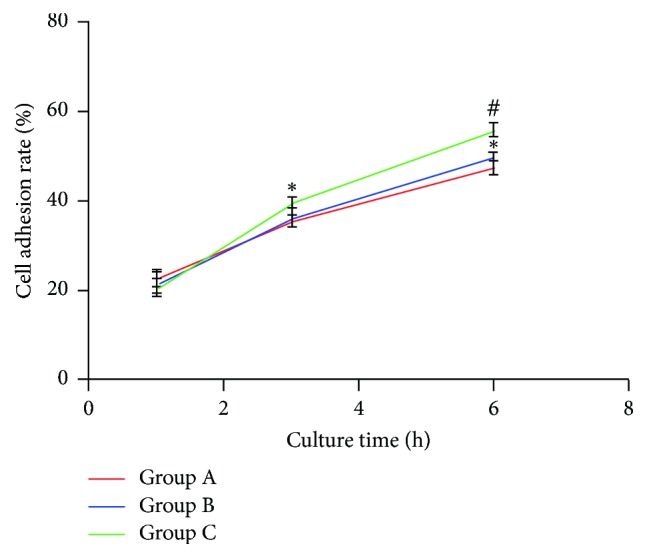
Cell adhesion rate of the P3HB4HB/(GEL + PVA) scaffolds on 1, 3, and 6 h. Group A: GEL : PVA = 0.6 g : 0.2 g; Group B: GEL : PVA = 0.4 g : 0.4 g; Group C: GEL : PVA = 0.2 g : 0.6 g. Compare to Group A, ^*∗*^*P* < 0.05; compare to Group B, ^#^*P* < 0.05.

As the incubation time prolonged, cell adhesion rate of the scaffolds in three groups increased. With the cells cultured for 6 hours, cell adhesion rate of Group C was higher than that of Groups A and Group B (Figure 7).

### 3.9. Cell Proliferation Assay

**Figure 8:**
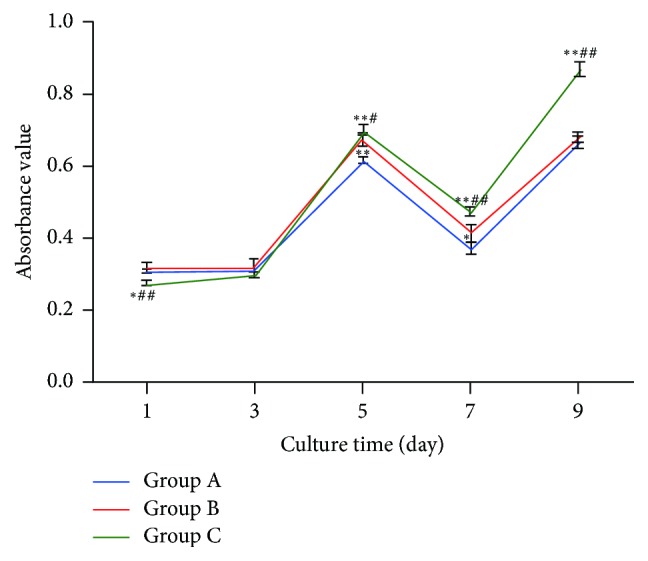
CCK-8 assay shows the proliferation of hBMSCs on days 1, 3, 5, 7, and 9  for the scaffold materials Group A: GEL : PVA = 0.6 g : 0.2 g; Group B: GEL : PVA = 0.4 g : 0.4 g; Group C: GEL : PVA = 0.2 g : 0.6 g. Compare to Group A: ^*∗*^*P* < 0.05; ^*∗∗*^*P* < 0.01; compare to Group B: ^#^*P* < 0.05; ^##^*P* < 0.01.

The absorbance values of the three groups of scaffolds increased as time progressed. The curves for the day 1–3 cultures were smooth and steep for the day 3–5 cultures and dropped with slow proliferation after days 5–7. The curves then rose after 9 days. The cell proliferation in Group C was greater than that in Group B, and Group B was greater than Group A (Figure 8).

### 3.10. Observation under Electron Microscopy

**Figure 9:**
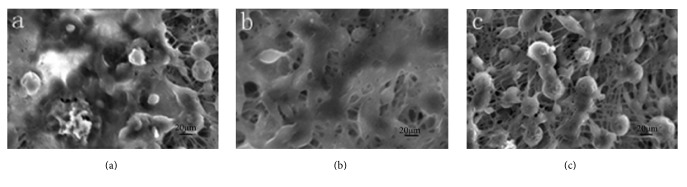
Observation of hBMSCs-P3HB4HB/(GEL + PVA) under electron microscope after* in vitro* culture for 7 days. (a) GEL : PVA = 0.6 g : 0.2 g; (b) GEL : PVA = 0.4 g : 0.4 g; (c) GEL : PVA = 0.2 g : 0.6 g.

After a 7-day* in vitro* culture of the cell-scaffold complex, fiber fusion and irregular membrane-like coverage were observed in Groups A and B, including the presence of round cells and lysis of some cells. In Group C, the nanofibrous filaments were clearly visible, and the cells were distributed on the scaffold surface. Additionally, most of the cells were round and the cells grew well, with a large amount of extracellular matrix secreted on the scaffold surface (Figure 9).

### 3.11. DAPI Fluorescent Staining

**Figure 10:**
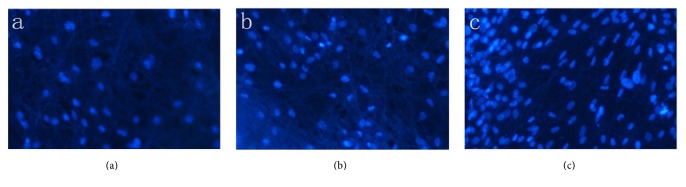
DAPI staining of hBMSCs-P3HB4HB/(GEL + PVA) after* in vitro* culture for 7 days. (a) GEL : PVA = 0.6 g : 0.2 g; (b) GEL : PVA = 0.4 g : 0.4 g; (c) GEL : PVA = 0.2 g : 0.6 g. The magnification is 200x.

The image of Group A was covered with a cloud-like substance. The nanofiber membrane was covered with gelatin in the form of a gel-like substance, and no obvious fibers and cells were observed, with no obvious pores. In Group B, the round blue cells and block-like substances were attached to the filaments, and the pores between the filaments were small and unevenly distributed. In Group C, a large number of cells were attached to the filaments, which were uniform in diameter and connected to each other to form a three-dimensional network (Figure 10).

### 3.12. Effect of Different Materials on the Differentiation Potential of the Stem Cells

**Figure 11:**
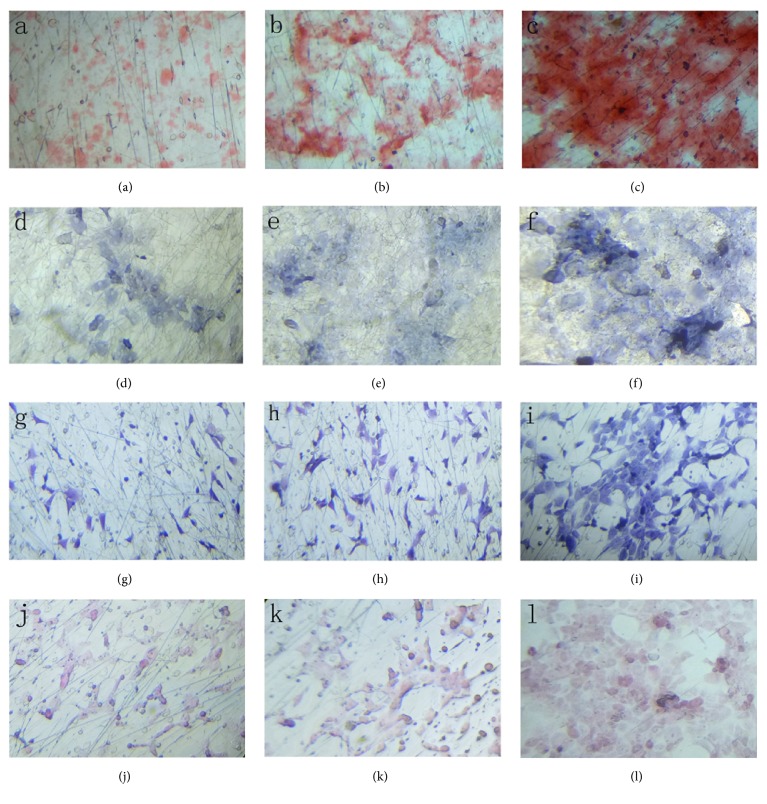
(a, d, g, j) GEL : PVA = 0.6 g : 0.2 g for alizarin red S, ALP, alcian blue, and safranin O staining; (b, e, h, k) GEL : PVA = 0.4 g : 0.4 g for alizarin red S, ALP, alcian blue, and safranin O staining; (c, f, i, l) GEL : PVA = 0.2 g : 0.6 g for alizarin red S, ALP, alcian blue, and safranin O staining. The magnification is 200x.

After culture induction, the hBMSCs on the three types of materials were able to maintain different degrees of osteogenic and chondrogenic differentiation. The specific staining of Group C was stronger than those of Groups A and B (Figure 11).

## 4. Discussion

There are many techniques to construct tissue engineering scaffolds, but most of the methods lack the ability to prepare 3D scaffolds with complex pore structures in a single step [19]. Electrostatic spinning has become a leading technology for the manufacture of tissue scaffolds, which can produce scaffolds with the desired morphology and porosity to suit the requirements of scaffold materials for tissue engineering. The single scaffold material has the advantages and disadvantages in terms of biological activity, hydrophilicity, and mechanical properties. In order to prepare the ideal scaffold material, it is inclined to use the different properties of a variety of materials to synthesize the properties of the single material, which is superior to the single material properties. In this experiment, by coaxial spinning gelatin, PVA, and P3HB4HB, we realized the composite construction of three kinds of materials. The experiment combines the advantages of the 3 materials to make up for the defects of different materials and give full play of their respective advantages.

An ideal scaffold material for bone tissue engineering can provide good three-dimensional space support for cell adhesion, proliferation, and differentiation. Additionally, the material should be completely degraded and absorbed after it performs its function, and the material degradation and the speed of osteogenesis should be well matched to prevent premature or late degradation [20]. In this study, the electrospun scaffold with gelatin : PVA = 2 : 6 (Group C) showed the highest porosity and a suitable pore size for cell growth. The high porosity can increase the contact area between the cells and the scaffold, and it can also promote tissue growth in the material. The size of spread MSCs was 20–50 *μ*m. The pore size of scaffold (25–50 *μ*m) was big enough to allow cellular migration and infiltration [21]. Researchers have also confirmed that the suitable porosity of scaffolds for the penetration of cells is within the range of 60–90% [18]. Scanning electron microscopy observations showed that the Group C scaffold formed an interconnected three-dimensional network with uniform fiber diameters and pore sizes and good interconnection among the pores.

The unique three-dimensional structure and extremely large specific surface area of nanofibers enable the electrospinning technology to have great potential in photocatalysts, capacitor electrodes, and tissue engineering [1]. A group of scaffolds containing more gelatin was soaked in 37°C liquid, nanofibers outer layer with gelatin dissolved, the weight of the scaffolds decreased, and the degradation increased. The degradation of C group with less gelatin content was relatively low. The CCK8 assay showed that the cells in the three groups of materials were well proliferated within 1–5 days. However, the curve dropped after 5–7 days and rose again after 7 days, which may have resulted from the good biocompatibility between the gelatin-PVA shell material and the cells, causing a rapid cell proliferation. After 5 days, however, the cells began to contact the P3HB4HB material in the core layer as the shell material gradually dissolved, suggesting that the volatile organic solvent in the core material may affect cell proliferation. Consequently, slower cell proliferation results. However, the cells would proliferate again as the residual solvent gradually reduced. Accordingly, in the composite tissue engineering scaffold constructed with synthetic and natural polymer materials, a coaxial electrospinning shell material with good biocompatibility can provide a buffer time for cell proliferation. The TEM results showed that the core-shell nanofibers were more likely to form in Group C, and the cell proliferation of Group C was greater than those of the other two groups. This study also demonstrated that the coaxial electrospinning technology could be applied to construct a composite scaffold structure with different materials; that is, a material with excellent electrospinning performance can be used to enhance a material with poor electrospinning performance, and a natural polymer material can be used to improve the hydrophilicity of a synthetic polymer material. Overall, a scaffold structure with tightly integrated core-shell layers can be obtained in which the layers do not negatively affect the function of each other.

The mechanical test of the composite fiber scaffold showed that the tensile strength and the modulus of elasticity increased as the gelatin content decreased. The reason is that the electrospinning performance of gelatin is poor, for the mixed solution with gelatin : PVA > 2 : 6, the electrospun fibers are covered by a large number of droplets, and the fibers are arranged irregularly and disorderly with varying diameters. For mechanical tensile, even a small force can cause a great deformation of the fiber. For this reason, diminished tensile strength and modulus of elasticity of elasticity are obtained with larger gelatin contents. Electron microscopy showed that larger gelatin contents led to a more disorderly arrangement of the fiber filaments. In addition, the fibers were more entangled or even intertwined. The comparison of the fiber membrane with the same length showed that the fibers in the membrane were longer and more entangled. In the tensile test, the membrane showed a greater elongation at fracture, resulting in a greater nominal strain fracture. With a decrease in the gelatin content, the modulus of elasticity was increased. This type of scaffold can be used for ligament tissue engineering. Thayer et al. [22] used collagen combined with electrospinning technology to prepare a ligament tissue engineering scaffold and found that its characteristics were similar to ligament. Further, Yang et al. [23] constructed a PCL/gelatin multilayer electrospun scaffold that can simulate the structure of tendon tissue.

The contact angle can reflect the hydrophilicity of a material, and the hydrophilicity directly affects the survival and proliferation of cells. Smaller contact angles indicate that a material is relatively more hydrophilic, and vice versa. It was found that there was no significant difference in the diameter of the three groups. With the decrease of gelatin content, the pore size of the scaffold becomes smaller and the porosity increases, and the contact angle increases. Three groups of fiber membrane surface roughness have the main indicators: roughness profile (*Ra*), the roughness height (*Rz*), and the distance between the contour peak line and the contour bottom line (*Ry*); Group C was greater than the Group B; Group B was larger than the Group A. Relative to A, Group B and Group C fiber membrane is more rough. Fiber membrane roughness can increase the membrane surface area and improve membrane hydrophobicity [24, 25]. The contact angle of Group C was larger than that of Group A and Group B. The hydrophilic functional group in gelatin improved the hydrophilicity of the fiber membrane. The experimental results showed that the hydrophilicities of the materials in the three groups were all less than 90°. The gelatin needs to be heated to dissolve, when the temperature reducing it will become a jelly again, so its spinnability is poor. Therefore in this experiment, we add polyvinyl alcohol into gelatin aqueous solution to improve the effect of electrospun spinning of gelatin. It is found that, with the increasing of PVA content in the solution, the electric spun is better, and the formation of core-shell structure is easier. The shell material is constructed with water-soluble polymeric material PVA in good electrospinning and can improve the poor electrospinning of gelatin, and the P3HB4HB coaxial electrospinning can improve the poor hydrophilicity of P3HB4HB. Furthermore, the mixed shell materials effectively addressed the problem of fast degradation of gelatin. Although the materials in Group A and B showed good hydrophilicity, both their porosity and cell growth were poor. Following* in vitro* culture with the Group C scaffold for 7 days, SEM and DAPI staining showed that a large number of cells adhered to the filaments and the cells grew well. The osteogenic and chondrogenic-specific induction and staining also showed that the best outcome occurred with Group C, indicating that the ratio of gelatin : PVA = 2 : 6 was optimal and did not affect the differentiation potential of the stem cells.

## 5. Conclusion

In this study, three-dimensional network scaffolds with high connectivity were generated using coaxial electrospinning technology to integrate the advantages of P3HB4HB, gelatin, and PVA. The best comprehensive performance of the composite scaffolds was obtained with a 0.2 g : 0.6 g mass ratio of gelatin to PVA. Indeed, the pore diameter, porosity, mechanical properties, and cell proliferation and differentiation were optimal, indicating that a composite scaffold with this mass ratio is an excellent material for cell growth and has great research and development potential, thus expanding the use of scaffold materials for bone tissue engineering. Further studies are desirable to investigate the* in vivo* repair of bone tissue with this composite scaffold.
